# Attenuation of fibrosis *in vitro *and *in vivo *with *SPARC *siRNA

**DOI:** 10.1186/ar2973

**Published:** 2010-04-01

**Authors:** Jiu-Cun Wang, Syeling Lai, Xinjian Guo, Xuefeng Zhang, Benoit de Crombrugghe, Sonali Sonnylal, Frank C Arnett, Xiaodong Zhou

**Affiliations:** 1 State Key Laboratory of Genetic Engineering and MOE Key Laboratory of Contemporary Anthropology, School of Life Sciences, Fudan University, 220 Handan Road, Shanghai 200433, PR China; 2 Division of Rheumatology and Clinical Immunogenetics, Department of Internal Medicine, The University of Texas Medical School at Houston, 6431 Fannin St, Houston, Texas 77030, USA; 3 Department of Pathology, Baylor College of Medicine, One Baylor plaza, Houston, Texas 77030, USA; 4 Department of Molecular Genetics, MD. Anderson Cancer Center, University of Texas, 1515 Holcombe Blvd, Houston, Texas 77030, USA

## Abstract

**Introduction:**

*SPARC *is a matricellular protein, which, along with other extracellular matrix components including collagens, is commonly over-expressed in fibrotic diseases. The purpose of this study was to examine whether inhibition of *SPARC *can regulate collagen expression *in vitro *and *in vivo*, and subsequently attenuate fibrotic stimulation by bleomycin in mouse skin and lungs.

**Methods:**

In *in vitro *studies, skin fibroblasts obtained from a Tgfbr1 knock-in mouse (TBR1^CA^; Cre-ER) were transfected with *SPARC *siRNA. Gene and protein expressions of the Col1a2 and the Ctgf were examined by real-time RT-PCR and Western blotting, respectively. In *in vivo *studies, C57BL/6 mice were induced for skin and lung fibrosis by bleomycin and followed by *SPARC *siRNA treatment through subcutaneous injection and intratracheal instillation, respectively. The pathological changes of skin and lungs were assessed by hematoxylin and eosin and Masson's trichrome stains. The expression changes of collagen in the tissues were assessed by real-time RT-PCR and non-crosslinked fibrillar collagen content assays.

**Results:**

*SPARC *siRNA significantly reduced gene and protein expression of collagen type 1 in fibroblasts obtained from the TBR1^CA^; Cre-ER mouse that was induced for constitutively active TGF-β receptor I. Skin and lung fibrosis induced by bleomycin was markedly reduced by treatment with *SPARC *siRNA. The anti-fibrotic effect of *SPARC *siRNA *in vivo *was accompanied by an inhibition of Ctgf expression in these same tissues.

**Conclusions:**

Specific inhibition of *SPARC *effectively reduced fibrotic changes *in vitro *and *in vivo*. SPARC inhibition may represent a potential therapeutic approach to fibrotic diseases.

## Introduction

Fibrosis is a general pathological process in which excessive deposition of extracellular matrix (ECM) occurs in the tissues. It is currently untreatable. Although therapeutic uses of some anti-inflammatory and immunosuppressive agents such as colchicine, interferon-gamma, corticosteroids and cyclophosphamide have been reported, many of these approaches have not proven successful [1-3]. Recently, SPARC (secreted protein, acidic and rich in cysteine), a matricellular component of the ECM, has been reported as a bio-marker for fibrosis in multiple fibrotic diseases, such as interstitial pulmonary fibrosis, renal interstitial fibrosis, cirrhosis, atherosclerotic lesions and scleroderma or systemic sclerosis (SSc) [4-9]. Notably, increased expression of SPARC has been observed in affected skin and circulation of patients with SSc [10,11], a devastating disease of systemic fibrosis, as well as in cultured dermal fibroblasts obtained from SSc skin [8,9].

SPARC, also called osteonectin or BM-40, is an important mediator of cell-matrix interaction [12]. Increasing evidence indicates that SPARC may play an important role in tissue fibrosis. In addition to its higher expression level in the tissues of fibrotic diseases, SPARC has shown a capacity to stimulate the transforming growth factor beta (TGF-β) signaling system [13]. Inhibition of SPARC attenuates the profibrotic effect of exogenous TGF-β in cultured human fibroblasts [14]. Moreover, in animal studies, SPARC-null mice display a diminished amount of pulmonary fibrosis compared with control mice after exposure to bleomycin, a chemotherapeutic antibiotic with a profibrotic effect [15]. These observations suggest that SPARC is a potential bio-target for anti-fibrotic therapy.

Recently, application of double-stranded small interfering RNA (siRNA) to induce RNA silencing in cells has been widely accepted in many studies of gene functions and potential therapeutic targets [16]. The selective and robust effect of RNAi on gene expression makes it a valuable research tool, both in cell culture and in living organisms. Unlike a gene knockout method, siRNA-based technology can easily silence the expression of a specific gene and is more feasible in practice, such as in disease therapy. Therefore, tissue-specific administration of the siRNA of candidate genes is currently being developed as a potential therapy in a great number of diseases, such as pulmonary diseases, ocular diseases, and others [17-19]. Our previous studies demonstrated that the overproduction of collagens in the fibroblasts obtained from SSc skin can be attenuated through SPARC silencing with siRNA. It suggested that application of SPARC silencing represents a potential therapeutic approach to fibrosis in SSc and other fibrotic diseases [20]. However, it is still unknown whether SPARC siRNA can improve fibrotic manifestations *in vivo*. The main purpose of the studies herein was to explore the feasibility of inhibition of SPARC with siRNA to counter fibrotic processes in a fibrotic mouse model *in vivo*. As a preliminary experiment in the *in vivo *studies, the fibroblasts cultured from a transgenic fibrotic model were used to assess the possibility and potential mechanisms of SPARC siRNA in attenuating the collagen expression *in vitro*. At the same time, the effects of SPARC siRNA to encounter fibrosis were compared with that of siRNA of CTGF, a well-known fibrotic marker. The fibrotic models used herein were the very popular bleomycin-induced skin and pulmonary fibrosis in mice. Subcutaneous injection and intratracheal instillation of siRNAs were used for tissue-specific treatments of skin and pulmonary fibrosis, respectively.

## Materials and methods

### Fibroblast cell lines from Tgfbr1 knock-in mouse

Constitutively activated Tgfbr1 mice, which recapitulated clinical, histological, and biochemical features of human SSc, have been reported previously [21]. They are termed TBR1^CA^; Cre-ER mice and harbor both the DNA for an inducible constitutively active TGFβ receptor I (TGFβRI) mutation targeted to the *ROSA *locus, and a Cre-ER transgene driven by a *Col1 *fibroblast-specific promoter. Fibroblasts were derived from skin biopsy specimens of these mice. The cultures were maintained in DMEM with 10% FCS and supplemented with antibiotics (50 U/ml penicillin and 50 μg/ml streptomycin). Fifth-passage fibroblast cells were seeded at a density of 5 × 10^5 ^cells in 25-cm^2 ^flasks and grown until confluence. Experiments were performed in triplicates.

### Transient transfection with siRNA in fibroblasts

Double-stranded ON-TARGET*plus *siRNAs of murine *SPARC *and *Ctgf *were purchased from Dharmacon, Inc. (Lafayette, CO, USA). The corresponding target sequences are 5'-GCACCACACGUUUCUUUG-3' for *SPARC *and 5'-GCACCAGUGUGAAGACAUA-3' for *Ctgf*, respectively. The culture medium in each culture flask with confluent fibroblasts was replaced with Opti-MEM I medium (Invitrogen, Carlsbad, CA, USA) without FCS and antibiotics. The fibroblasts were incubated for 24 hours and transfected with *SPARC *siRNA or Ctgf siRNA in a concentration of 100 nmol/L, using Dharma*FECT*™ 1 siRNA Transfection Reagent (Dharmacon). Fibroblasts with Non-Targeting siRNA (Dharmacon) treatment were used as negative controls. The non-targeting siRNA was characterized by genome-wide microarray analysis and found to have minimal off-target signatures to human cells. It targets firefly luciferase (U47296). After 24 hours, the culture medium was replaced with DMEM. The cells transfected with siRNA were examined after 72 hours of transfection and used for RNA and protein expression analysis. The experiments were performed in triplicates.

### Animal models of fibrosis

C57BL/6 mice of about 20 grams were purchased from Jackson Laboratory (Bar Harbor, ME, USA). Bleomycin from Teva Parenteral Medicines Inc. (Irvine, CA, USA) was dissolved in saline and used in the mice at a concentration of 3.5 units/kg. Pulmonary fibrosis was induced in these mice with one time intratracheal instillation of bleomycin. For dermal fibrosis, female C57BL/6 mice at six weeks (weighing about 20 g) were treated daily for four weeks with local subcutaneous injection of 100 μl bleomycin in the shaved lower back. Four mice were used in each group. The animal protocols were approved by the Center for Laboratory Animal Medicine and Care in the University of Texas Health Science Center at Houston, the Institutional Animal Use and Care Committee of M.D. Anderson Cancer Center, and Fudan University, China.

### Administration of siRNAs *in vivo*

For pulmonary fibrosis, 3 μg of siRNA for *in vivo *use (siSTABLE, Dharmacon), mixed with Dharma*FECT*™ 1 siRNA Transfection Reagent, was administrated intratracheally in 60 μl on Days 2, 5, 12 after bleomycin treatment. In addition, the siGLO Green transfection indicator (Dharmacon), a fluorescent RNA duplex was used for evaluating distribution of intratracheally injected siRNA. Twenty-four hours after injection, lung tissues were obtained for processing slides using a cryo-microtomy. All the mice were sacrificed on Day 23 after anesthesia, and the lung samples were collected. The left lungs were fixed by 4% formalin and used for further histological analysis. The right lungs were minced to small pieces and divided into two parts, one for RNA extraction and one for collagen content analysis.

For dermal fibrosis, the above siRNAs were injected into the same area as that of bleomycin three hours after bleomycin treatment and continued for four weeks. The mice were sacrificed on Day 29 and the skin samples were collected. Saline was used as a negative control in both fibrosis studies.

### Determination of gene expression by quantitative RT-PCR

Total RNA from each cell line was extracted from the cultured fibroblasts using RNeasy Mini Kit (Qiagen, Valencia, CA, USA). For mice lung and skin tissues, the minced samples were homogenized in lysis solution (Sigma-Aldrich, St. Louis, MO, USA) with a blender. Then total RNA was extracted using GenElute™ Mammalian Total RNA Miniprep Kit (Sigma-Aldrich). Complementary DNA (cDNA) was synthesized using MultiScribe™ Reverse Transcriptase (Applied Biosystems, Foster city, CA, USA). Quantitative real-time RT-PCR was performed using an ABI 7900 Sequence Detector System (Applied Biosystems). The specific primers and probes for each gene (*Col1a2, Col3A1, Ctgf, SPARC* and *Ccl2*) were purchased from the Assays-on-Demand product line (Applied Biosystems). Synthesized cDNAs were mixed with primers/probes in 2 × TaqMan universal PCR buffer and then assayed on an ABI 7900 sequence detector. The data obtained from the assays were analyzed with SDS 2.2 software (Applied Biosystems). The expression level of each gene in each sample was normalized with *Gapdh *transcript level.

### Western blot analysis

The lysis buffer for Western blot analysis consisted of 1% Triton X-100, 0.5% Deoxycholate Acid, 0.1% SDS, 1 mM EDTA in PBS and proteinase inhibitor cocktail from Roche (Basel, Switzerland). The cellular lysates extracted from the cultured fibroblasts were used for protein assays. The protein concentration was determined by a spectrophotometer using Bradford protein assay kit (Bio-Rad Laboratories, Hercules, CA, USA). Equal amounts of protein from each sample were subjected to sodium dodecyl sulfate-polyacrylamide gel electrophoresis. Resolved proteins were transferred onto PVDF membranes and incubated with respective primary antibodies, including anti-type I collagen antibody (Biodesign International, Saco, ME, USA), anti-CTGF antibody (GeneTex Inc, San Antonio, TX, USA), and anti-SPARC antibody (R&D Systems Inc, Minneapolis, MN, USA). Mouse β-actin (Alexis Biochemicals, San Diego, CA, USA) was used as an internal control. The secondary antibody was peroxidase-conjugated anti-rabbit, anti-goat, or anti-mouse IgG. Specific proteins were detected by chemiluminescence using an enhanced chemiluminescence system (Amersham, Piscataway, NJ, USA). The intensity of the bands was quantified using ImageQuant software (Molecular Dynamics, Sunnyvale, CA, USA).

### Determination of collagen content

Non-crosslinked fibrillar collagen in lung samples and skin samples was measured using the Sircol colorimetric assay (Biocolor, Belfast, UK). Minced tissues were homogenized in 0.5 M acetic acid with about 1:10 ratio of pepsin (Sigma-Aldrich). Tissues were weighted, and then incubated overnight at 4°C with vigorous stirring. Digested samples were centrifuged and the supernatant was used for the analysis with the Sircol dye reagent. The protein concentration was determined using Bradford protein assay kits and the collagen content of each sample was normalized to total protein.

### Histological analysis

The tissue samples of both lung and skin were fixed in 4% formalin and embedded in paraffin. Sections of 5 μm were stained either with hematoxylin and eosin (HE) and Masson's trichrome.

### Statistical analysis

Results were expressed as mean ± SD). The difference between different conditions or treatments was assessed by Student's t-test. A *P*-value of less than 0.05 was considered statistically significant.

## Results

### Gene and protein expression of *Col1a2, Ctgf *and *SPARC *in the fibroblasts from TBR1^CA^; Cre-ER mice with and without transfection of siRNAs of *SPARC *or Ctgf

**Figure 1:**
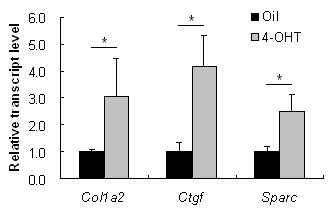
**Comparison of gene expression between the fibroblasts of TBR1^CA^; Cre-ER mice injected with oil and 4-OHT**. The expression level of each gene in the fibroblasts of TBR1^CA^; Cre-ER mice injected with oil was normalized to 1. Bars show the mean ± SD results of analysis of three independent experiments performed in triplicate. *, *P *< 0.05.

**Figure 2:**
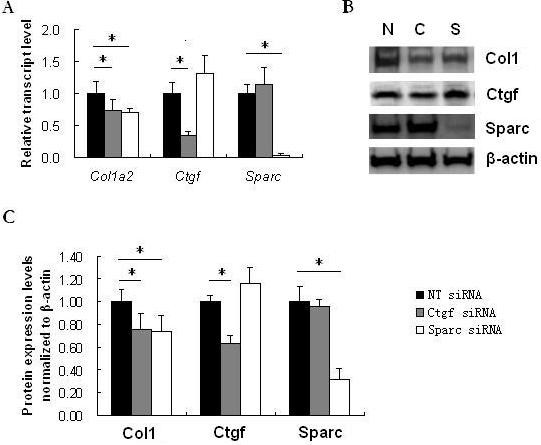
**Gene and protein expression in original and siRNA treated fibroblasts from TBR1CA; Cre-ER mice injected with 4-OHT**. **(A) **Relative transcript levels of Col1a2, Ctgf, and *SPARC *in cultured fibroblasts transfected with non-targeting siRNA (NT siRNA), Ctgf siRNA and *SPARC *siRNA. The expression level of each gene in the fibroblast lines with NT siRNA transfection was normalized to 1. *, *P *< 0.05. **(B) **Western blot analysis of type I collagen (COL1), CTGF, and SPARC in the fibroblasts from constitutively active Tgfbr1 mice transfected with NT siRNA, Ctgf siRNA or *SPARC *siRNA. N, non-targeting siRNA transfected fibroblasts; C, Ctgf siRNA transfected fibroblasts; S, *SPARC *siRNA transfected fibroblasts. **(C) **Densitometric analysis of Western blots for protein level of COL1, CTGF, and SPARC. Compared to non-targeting siRNA treatment, Ctgf siRNA or *SPARC *siRNA transfected fibroblasts showed significant reduction of COL1 (*P *= 0.015 or 0.009 respectively). Significant reduction of CTGF (*P *= .002) by Ctgf siRNA and SPARC (*P *= 0.0004) by *SPARC *siRNA were also shown. Bars show the mean ± SD results of analysis of three independent experiments performed in triplicate. *, *P *< 0.05.

As measured by quantitative real-time RT-PCR, the transcripts of *Col1a2, Ctgf *and *SPARC *showed increased expression in the fibroblasts from TBR1^CA^; Cre-ER mice injected with 4-OHT, in which Tgfbr1 was constitutively active, compared with those in the cells from TBR1^CA^; Cre-ER mice injected with oil (Figure 1). The fold-changes of each gene in 4-OHT-injected TBR1^CA^; Cre-ER mice fibroblasts were 3.06 ± 1.42 for *Col1a2 *(*P *= 0.050), 4.15 ± 1.18 for *Ctgf *(*P *= 0.049), and 2.49 ± 0.63 for *SPARC *(*P *= 0.017), respectively. To study whether inhibition of *SPARC *induced a reduction of collagen in the fibroblasts from constitutively active Tgfbr1 mice, we transfected *SPARC *siRNA into cultured fibroblasts obtained from TBR1^CA^; Cre-ER mice injected with 4-OHT. Ctgf is a down-stream gene in the TGF-β pathway [22-25], and inhibition of Ctgf reduced expression of the fibrotic effect of TGF-β [26]. We used Ctgf siRNA as a positive control for inhibition of Ctgf and collagen expression. Transfection efficiency of siRNAs into fibroblasts was measured using fluorescent RNA duplex si*GLO *Green transfection indicator (Dharmacon) and was determined to be over 80%. The gene expression levels from the Non-Targeting siRNA treated fibroblasts were compared with those from saline-treatment fibroblasts, and no significant differences were found (1.05 ± 0.18-folds for *Col1a2*, 1.14 ± 0.16-folds for *Ctgf*, and 1.12 ± 0.12-folds for *SPARC*). Therefore, in the following *in vitro *study, fibroblasts with Non-Targeting siRNA treatment were used as negative controls. Seventy-two hours after *SPARC *siRNA or Ctgf siRNA transfection, significant reductions of *SPARC *(95%) by *SPARC *siRNA and *Ctgf *(64%) by Ctgf siRNA were observed in the fibroblasts (Figure 2A). In parallel, *Col1a2 *showed decreased expression in both siRNA transfected fibroblasts (27% and 29% decrease with *P *< 0.05 for Ctgf siRNA and *SPARC *siRNA, respectively) (Figure 2A). Western blot analysis showed a similar level of protein reduction of type I collagen by either *SPARC *siRNA or Ctgf siRNA treatment. As illustrated in Figure 2B, C, both *SPARC *siRNA and Ctgf siRNA showed significant attenuation of collagen type I in the fibroblasts (*P *= 0.009 or 0.015, respectively). CTGF and SPARC protein levels also were reduced by their corresponding siRNAs (*P *= 0.002 and 0.0004, respectively).

### siRNAs of *SPARC *and Ctgf ameliorated fibrosis in skin and reduced inflammation in lungs induced by bleomycin

**Figure 3:**
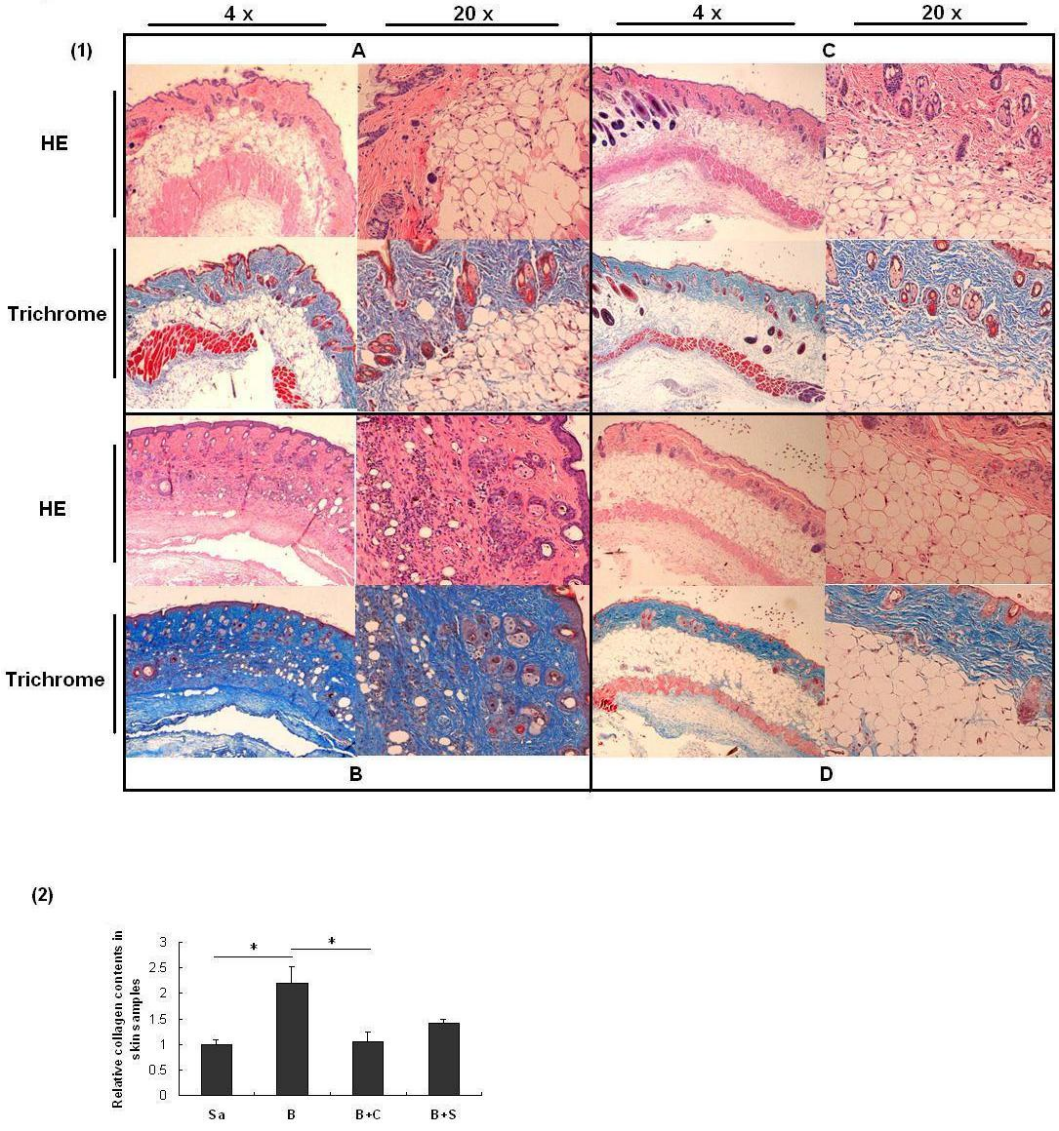
**Examination of skin tissues**. **(1) **Representative histological analysis of HE and Trichrome stain of mouse skin with different treatments for four weeks in low (4 ×) and high magnifications (20 ×). Four mice were used for each group. **A**. Injection with saline (negative control) only; **B**. Injection with bleomycin only; **C**. Injection with bleomycin and treatment with *SPARC *siRNA; **D**. Injection with bleomycin and treatment with Ctgf siRNA. **(2) **Collagen contents in skin samples with different treatments. The collagen content in the skin sample from saline treated mice was normalized to 1. Treatments: Sa, saline; B, bleomycin; B + C, bleomycin and Ctgf siRNAs; B + S, bleomycin and *SPARC *siRNA. *P *< 0.05.

HE stains of mouse skin tissues (Figure 3-1) showed that four-week injections of bleomycin induced significant fibrosis in skin where the fat cells were replaced by fiber bundles (Figure 3-1B, compared with normal skin injected with saline only (Figure 3-1A). Bleomycin-injected skin treated with *SPARC *siRNA or Ctgf siRNA showed that most of the fat cells still existed in the dermis without prominent fiber bundles (Figure 3-1C, D). Masson's trichrome staining of the samples also showed the same results. Notably, increased hair follicles were inconsistently seen in Ctgf siRNA- and *SPARC *siRNA-treated bleomycin-induced skins.

**Figure 4:**
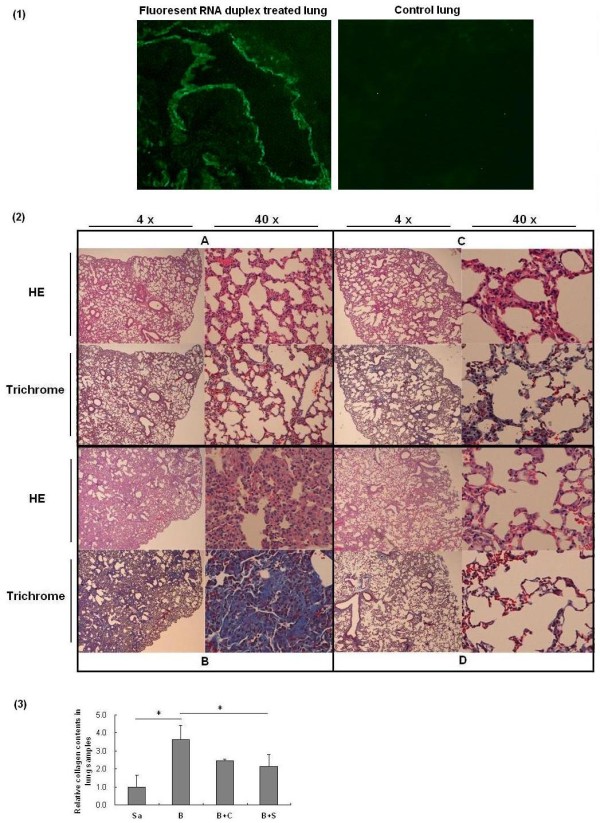
**Examination of lung tissues**. **(1) **The lung tissue staining for intratracheally injected fluorescent siRNA. Intense fluorescence was observed within epithelial cells of bronchi and bronchioles, and weak fluorescence was detected in the parenchyma. **(2) **Representative histological features of HE and Trichrome stain of mouse lung samples with different treatments intratracheally in low (4 ×) and high magnifications (40 ×). Four mice were used for each group. **A**. Injection with saline (negative control) only; **B**. Injection with bleomycin only on Day 0; **C**. Injection with bleomycin on Day 0 and *SPARC *siRNA on Days 2, 5, and 12; **D**. Injection with bleomycin on Day 0 and Ctgf siRNA on Days 2, 5, and 12. **(3) **Collagen contents in lung samples with different treatments. The collagen content in the lung sample from saline treated mice was normalized to 1. Four mice were used for each group. Treatments: Sa, saline; B, bleomycin; B + C, bleomycin and Ctgf siRNAs; B + S, bleomycin and *SPARC *siRNA. *, *P *< 0.05.

The lung distribution of intratracheally injected fluorescent siRNA showed that intense fluorescence was distributed within epithelial cells of bronchi and bronchioles, and only weak fluorescence was detected in the parenchyma (Figure 4-1).

**Figure 5:**
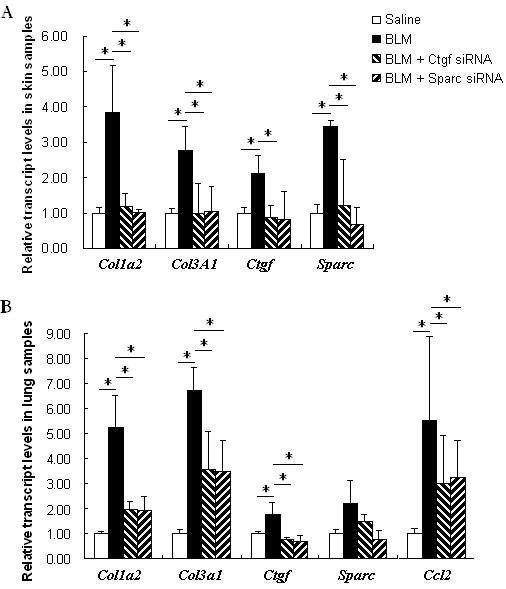
**Gene expression in skin (A) or lung samples (B) with different treatments**. Four mice were used for each treatment. The relative transcript levels of *Col1a2, Col3a1, Ctgf, SPARC *and *Ccl2 *in siRNA-treated or untreated bleomycin-induced skins or lungs, respectively. The expression level of each gene in the skin or lung sample from saline treated mice was normalized to 1. Treatments: Saline; BLM (bleomycin); BLM + Ctgf siRNA and BLM + *SPARC *siRNA. *, *P *< 0.05.

HE stain of mouse lung tissues (Figure 4-2) showed a significant disruption of the alveolar units and infiltration of inflammatory cells in the lungs induced by bleomycin (Figure 4-2B), compared with saline injection (Figure 4-2A). However, after treatment with Ctgf siRNA or *SPARC *siRNA, the disruption of the alveoli was improved with less infiltrating inflammatory cells (Figure 4-2C, D). In addition, both siRNA treatments showed a significant reduction of gene expression of *Ccl2*, an active biomaker of inflammation, which was up-regulated in bleomycin stimulated mice (Figure 5B).

### siRNAs of *SPARC *and Ctgf reduced the collagen contents in bleomycin-induced mouse skin and lung tissues

To further evaluate anti-fibrotic effects of siRNAs on the fibrogenesis of skin and lung, the collagen content was measured in the collected dermal and pulmonary samples. Quantification of total collagen in skin samples with the Sircol assay showed a 2.2-fold increase in bleomycin-induced skin compared with saline-injected skin (*P *= 0.050). Ctgf siRNA treatment reduced the collagen content significantly to 47.6% (*P *= 0.028) of that in bleomycin-induced skin, and *SPARC *siRNA treatment reduced the collagen content to 64.6% (*P *= 0.077) but not very significantly (Figure 3-2). The difference of collagen reduction (*P *= 0.076) between *SPARC *siRNA treatment and Ctgf siRNA treatment was not very significant might due to the small sample size.

The siRNA treatments also showed a reduction of collagen in the lung tissues of bleomycin-induced mice (Figure 4-2). In bleomycin-induced mice, collagen content of lung tissues was 3.6-fold higher than that in saline-injected control mice (*P *= 0.014). In *SPARC *siRNA treated mice that also were bleomycin-induced, the collagen content of lung tissues was significantly reduced to 58% (*P *= 0.019) of that in bleomycin-induced mice without siRNA treatment. Ctgf siRNA also reduced the collagen content to a quite low level (68% of that without siRNA treatment) but without significance (*P *= 0.128). Further, no significant difference of collagen content was found between *SPARC *siRNA treatment and Ctgf siRNA treatment in bleomycin-injured lungs (*P *= 0.277).

### siRNAs of *SPARC *and Ctgf attenuated over-expression of collagen and other fibrotic ECM genes induced by bleomycin in skin and lung tissues

Bleomycin injection induced an up-regulation of the *Col1a2*, *Col3a1, Ctgf *and *SPARC *gene in both skin (Figure 5A, *P *= 0.028, 0.016, 0.049 and 0.0005, respectively) and lung tissues (Figure 5B, *P *= 0.015, 0.005, 0.041 and 0.056, respectively) of the mice significantly or marginal significantly. However, in Ctgf siRNA or *SPARC *siRNA treated mice skin that also received bleomycin injection, the expression of the *Col1a2 *and *Col3a1 *appeared to be normal in skin tissues (Figure 5A, *P *= 0.025 and 0.003 for each gene in Ctgf siRNA treatment, and *P *= 0.031 and 0.010 in *SPARC *siRNA treatment), and were significantly improved in lung tissues (about 2.7-fold reduction for *Col1a2 *and 1.9-fold reduction for *Col3a1*, compared to bleomyin-injected mice without siRNA treatment, *P *< 0.05 for both) (Figure 5B). In addition to collagen gene expression, the *Ctgf *and the *SPARC *expression were significantly or marginal significantly reduced by *SPARC *siRNA and Ctgf siRNA treatment, respectively (Figure 5B). In detail, compared to bleomycin-induced skin and lungs, *SPARC *siRNA normalized *Ctgf *expression in both skin and lungs (2.6-fold reduction in both with *P *= 0.100 and 0.039, respectively). Similarly, Ctgf siRNA also reduced *SPARC *expression in skin and lungs (2.9-fold and 1.5-fold reduction with *P *= 0.044 and 0.102, respectively).

## Discussion

Although fibrosis is usually an irreversible pathological condition, targeting underlying molecular effectors may reverse an active status of the fibrotic process, and subsequently inhibit fibrosis. The TGF-β signaling pathway is associated with active fibrosis [22,23]. It begins with the binding of the TGF-β ligand to the TGF-β type II receptor, which catalyses the phosphorylation of the type I receptor on the cell membrane. The type I receptor then induces the phosphorylation of receptor-regulated SMADs (R-SMADs) that bind the coSMAD. The phosphorylated R-SMAD/coSMAD complex enters the nucleus acting as transcription factors to regulate target gene expression [22,23]. CTGF (connective tissue growth factor) is a down-stream gene that can be activated by the TGF-β signaling pathway [23,24]. Activation of CTGF is associated with potent and persistent fibrotic changes in the tissues, which is typically represented as accumulation of the ECM components including collagens [24,25]. SPARC also is involved in TGF-β signaling. It was reported that SPARC stimulated Smad2 phosphorylation and Smad2/3 nuclear translation in lung epithelial cells [27]. Recently, while examining SPARC regulatory role on the ECM components in human fibroblasts using linear structure equations, we demonstrated that SPARC positively controlled the expression of CTGF [26]. Although down-regulation of CTGF has been employed in treating fibrotic conditions [28], application of SPARC inhibition in attenuation of a fibrotic process in a therapeutic animal model has not been reported.

The studies described here first utilized the fibroblasts obtained from the TBR1^CA^; Cre-ER mice that were induced for constitutively active TGF-β receptor I. After transfection of *SPARC *siRNA, the fibroblasts showed a decreased expression of Col1a2 that was originally over-expressed in the TBR1^CA^; Cre-ER mice (Figure 2). This phenomenon suggests that *SPARC *inhibition may interrupt fibrotic TGF-β signaling, which generally induces collagen production. Although the specific mechanism for this suppression is unclear, multiple previous studies have demonstrated a mutual regulatory relationship between SPARC and TGF-β signaling [14,26,29]. This notion also is supported by the observation of an over-expression of *SPARC *in the fibroblasts of the TBR1^CA^; Cre-ER mice (Figure 1). It should be noted that the Ctgf expression in the fibroblasts was not reduced upon *SPARC *inhibition. These results appear to contradict our previous report of parallel inhibition of SPARC and CTGF expression in human fibroblasts by SPARC siRNA [14]. A possible explanation is that over-expressed Ctgf from constitutively activated TGF-β signaling in these fibroblasts may confer resistance to a down-regulatory effect from *SPARC *siRNA. However, such resistance appeared to have limited influence on any down-regulatory effect of *SPARC *siRNA on collagen type 1, which suggests that CTGF is not a sole contributor to TGF-β signaling-associated fibrosis.

Bleomycin induced fibrosis in mice usually occurs after inflammation in which TGF-β is up-regulated [30]. Our *in vivo *application of *SPARC *siRNA demonstrated that inhibition of *SPARC *significantly reduced fibrosis in skin and lungs induced by bleomycin. In the treatment of skin fibrosis, *SPARC *siRNAs reduced fiber bundles accumulated in the dermis with less mononuclear cell infiltrates (Figure 3-1). In addition to histological changes, the thickness of bleomycin-induced skin treated with *SPARC *siRNA showed over 50% reduction compared to that without *SPARC *siRNA treatment (data not shown). The changes of tissue fibrotic level further were confirmed with significantly decreased collagen gene expression (Figure 5A). Non-crosslinked fibrillar collagen in the skin tissues also showed an average of 35.4% reduction after *SPARC *siRNA treatment (Figure 3-2).

In the treatment of lungs, *SPARC *siRNA reduced the disruption and inflammatory cells of the alveoli induced by bleomycin (Figure 4-2), which was accompanied with attenuated gene expression and protein content of collagens as compared to that without siRNA treatment (Figures 5B and 4-3). In addition, a significant reduction of the Ccl2 expression in the siRNA-treated lung tissues also suggests an improvement of inflammation supporting the findings in histological staining. These observations are consistent with previous reports on SPARC-null mice that exhibited attenuation of inflammation and fibrosis in kidneys [31]. While precise mechanism of these changes is still unknown, increased expression of SPARC was reported to correlate with the levels of inflammatory markers [32,33]. It is likely that *SPARC *inhibition altered composition of microenvironment of the tissues that may restrain inflammatory response. On the other hand, much higher levels of gene expression of *Col1A2 *and *Col3A1*, and protein content of collagen were observed in bleomycin-induced lung tissues when they were compared to that in skin tissues (5.2-fold, 6.7-fold and 3.6-fold increase vs. 3.8-fold, 2.8-fold and 2.2-fold increase, respectively), which suggested that tissue damage and fibrosis in lung might be more severe than that in skin. In this case, treatment of bleomycin-induced lung damage might present a bigger challenge than that of skin, and the siRNA treatment through intratracheal instillation may be in need of further optimization. These notions were supported by similar findings in the treatment with the Ctgf siRNA, a positive control for anti-fibrotic effects.

Nevertheless, *SPARC *inhibition showed a clear anti-fibrotic effect in bleomycin-induced skin and lung tissues. Notably, these changes were accompanied with a significant down regulation of *Ctgf *that paralleled with *Ctgf *up-regulation in bleomycin-induced tissues. Thus, *SPARC *might regulate the collagen expression through affecting the expression of Ctgf, a TGF-β activity biomarker and down-strain gene, in bleomycin-induced mice. These observations combined with the results of anti-fibrotic effects of *SPARC *siRNA in fibroblasts of the Tgfbr1 knock-in mouse further support a mutually regulatory relationship between SPARC and TGF-β signaling.

## Conclusions

Studies described here consistently demonstrated that inhibition of *SPARC *with siRNA significantly reduced collagen expression in both *in vitro *transgenic Tgfbr1 fibroblast model and *in vivo *bleomycin-induced fibrotic mouse models. This is the first attempt to examine the anti-fibrotic effects of SPARC inhibition using siRNA with tissue-specific administration in skin and lungs *in vivo*. The results obtained from these studies provide favorable evidence that SPARC may be used as a bio-target for application of anti-fibrosis therapies.

## Abbreviations

Ccl2: Chemokine (C-C motif) ligand 2, also known as monocyte chemotactic protein-1 (MCP-1); Col: collagen; Ctgf: connective growth factor; ECM: extracellular matrix; HE: hematoxylin and eosin; siRNA: small interfering RNA; *SPARC: *secreted protein, acidic and rich in cysteine; SSc: systemic sclerosis; TGF-β: transforming growth factor beta;

## Competing interests

The authors are preparing a patent application for SPARC inhibition in the treatment of fibrosis. The authors declare that they have no other competing interests.

## Authors' contributions

WJ carried out the animal studies and most of the molecular studies. LS carried out tissue histological examination. GX and ZX carried out molecular studies. CB and SS provided fibroblasts from TBR1^CA^; Cre-ER mice. FA participated in coordination and helped to draft the manuscript. ZX carried out animal studies and participated in study design and drafting of the manuscript. All authors read and approved the final manuscript.
